# National survey of intensive care trainees' experience of and training in tracheostomy and laryngectomy management

**DOI:** 10.1186/2197-425X-3-S1-A678

**Published:** 2015-10-01

**Authors:** L Paton, T Monro-Somerville, A Gibson, L Gemmell

**Affiliations:** Victoria Infirmary, Glasgow, United Kingdom; Royal Infirmary, Edinburgh, United Kingdom

## Introduction

Recent studies in the United Kingdom [1],[2] have highlighted serious shortfalls in the care of patients with a tracheostomy or laryngectomy. Such patients potentially require time-critical, decisive airway management, likely involving resident intensive care clinicians. However, previous regional studies identified critical care trainees as inadequately trained and experienced to confidently manage airway emergencies in neck breathers [3].

## Objectives

We surveyed Scottish intensive care trainees to ascertain whether such deficits are widespread and persistent.

## Methods

Trainees were invited to complete a web-based questionnaire on three occasions in June 2014. Responses in the form of ticked boxes or free text were collated and analysed for trends.

## Results

Ninety-nine trainees replied, of whom 96 were based in anaesthesia, one in emergency medicine and two in allied medical specialties. Alarmingly, 70 respondents (71%) had been involved in managing an airway emergency in a patient with a tracheostomy or laryngectomy, 63 (90%) on a critical care unit and 40 (57%) on a general ward. Deficits in staff training and equipment were identified as contributory by 28 (40%) and 10 (14%) respondents, respectively. Most trainees (68%) felt quite confident in caring for neck breathers, with confidence increasing as training progressed. However, the majority of trainees accrued such confidence through exposure to these patients on theatre lists (80%). Only a minority had participated in simulated emergencies (33%) or a formal training course (14%). Almost half (49%) rated their training as deficient or absent and the vast majority (96%) felt there was a place for more formal tuition in caring for patients with a tracheostomy or laryngectomy.

## Conclusions

Our findings suggest that we are failing to prepare trainees for the challenges of managing airway emergencies in neck breathers. Divergence of anaesthesia and intensive care training in the UK may further compound this problem by reducing exposure to controlled subglottic airway management in theatre. The Scottish Intensive Care Society Trainee Committee has sought to address these deficits in training and reduce the burden of avoidable harm by incorporating sessions on tracheostomy management into its educational programme.
